# Exploiting Multi-Omics Profiling and Systems Biology to Investigate Functions of TOMM34

**DOI:** 10.3390/biology12020198

**Published:** 2023-01-28

**Authors:** Ekaterina V. Poverennaya, Mikhail A. Pyatnitskiy, Georgii V. Dolgalev, Viktoria A. Arzumanian, Olga I. Kiseleva, Ilya Yu. Kurbatov, Leonid K. Kurbatov, Igor V. Vakhrushev, Daniil D. Romashin, Yan S. Kim, Elena A. Ponomarenko

**Affiliations:** 1 Institute of Biomedical Chemistry, Moscow 119121, Russia; 2 Faculty Of Computer Science, National Research University Higher School of Economics, Moscow 101000, Russia

**Keywords:** *TOMM34*, gene knockout, HepG2, multi-omics profiling, pathway analysis, de novo network enrichment

## Abstract

**Simple Summary:**

Many human genes still have poorly understood functions despite often being connected to severe diseases. One of such genes is called *TOMM34*. This gene encodes a protein that helps to correctly shape and transport other proteins to mitochondria, which are organelles that supply the cell with energy. However, *TOMM34* has been associated with many diseases including cancer and neurodegeneration, which suggests that this gene has other functions. To investigate *TOMM34*’s possible functions, we engineered cells that have *TOMM34* gene deleted so that no protein product is produced. We then measured all ribonucleic acids, proteins, and metabolites present in both normal and edited cells. By comparing levels of individual molecules between normal and edited cells, we can understand which molecules and networks of molecules are disrupted in edited cells in comparison to normal cells. This lets us make predictions about *TOMM34*’s functions. Our results confirm previous information that *TOMM34* is important for mitochondrial functioning, but we also report for the first time that *TOMM34* is connected to processes of purine metabolism, DNA replication, and repair, as well as protein degradation and cellular signaling.

**Abstract:**

Although modern biology is now in the post-genomic era with vastly increased access to high-quality data, the set of human genes with a known function remains far from complete. This is especially true for hundreds of mitochondria-associated genes, which are under-characterized and lack clear functional annotation. However, with the advent of multi-omics profiling methods coupled with systems biology algorithms, the cellular role of many such genes can be elucidated. Here, we report genes and pathways associated with *TOMM34*, Translocase of Outer Mitochondrial Membrane, which plays role in the mitochondrial protein import as a part of cytosolic complex together with Hsp70/Hsp90 and is upregulated in various cancers. We identified genes, proteins, and metabolites altered in *TOMM34*^-/-^ HepG2 cells. To our knowledge, this is the first attempt to study the functional capacity of *TOMM34* using a multi-omics strategy. We demonstrate that *TOMM34* affects various processes including oxidative phosphorylation, citric acid cycle, metabolism of purine, and several amino acids. Besides the analysis of already known pathways, we utilized de novo network enrichment algorithm to extract novel perturbed subnetworks, thus obtaining evidence that *TOMM34* potentially plays role in several other cellular processes, including NOTCH-, MAPK-, and STAT3-signaling. Collectively, our findings provide new insights into *TOMM34*’s cellular functions.

## 1. Introduction

Despite the ever-increasing volume of data from biological experiments, a significant portion of genes in the human genome remains poorly characterized from a functional perspective [1]. This holds especially true for mitochondrial and mitochondria-associated genes, hundreds of which lack clear functions amid the existence of dozens of severe mitochondrial disorders with unknown genetic basis [2].

One such under-characterized gene is Translocase of Outer Mitochondrial Membrane 34, *TOMM34*. This gene is located on chromosome 20 and encodes a single protein isoform, mitochondrial import receptor subunit TOMM34, which consists of 309 amino acids [3]. According to the Human Protein Atlas [4], this protein is expressed at a relatively low level across all human tissues while being highly abundant in the testis.

Protein TOMM34 was initially discovered based on having a shared tetratricopeptide repeat motif with protein subunits of Translocase of outer membrane (TOM) complex [3]. This complex plays important role in mitochondria protein import and its activity is regulated by at least four different cytosolic signaling systems by phosphorylation [5]. In the initial study, TOMM34 was found to be present in mitochondrial membrane fraction where it co-localized with another subunit of the TOM complex, TOMM20 [3]. Antibodies raised against TOMM34 affected mitochondrial import of several proteins, thus supporting the role of TOMM34 in mitochondrial protein import. However, a following study that utilized a more sensitive antibody identified TOMM34 primarily in cytosol and only faintly in mitochondrial outer membrane [6]. Despite this, the necessity of TOMM34 for the correct import of some of mitochondrial proteins was confirmed. Two additional studies also provided evidence that refuted the association of TOMM34 with the TOM complex. In the first study, TOMM34 was shown to be mostly cytosolic and interact with mature portions of precursor mitochondrial proteins in contrast to TOM20, which binds to the leader sequences of precursor proteins [7]. In the second study, using yeast two-hybrid (Y2H) technique, authors failed to detect any interaction between TOMM34 and subunits of the TOM complex [8]. In parallel, TOMM34 was found to interact with a chaperone Hsp90 [9]. This prompted the researchers to explore the hypothesis that TOMM34 acts as a cochaperone of Hsp90, whose role in mitochondrial protein import was well established [10]. Indeed, TOMM34 was confirmed to form a large cytosolic complex together with both Hsp90 and Hsp70, where it was required for the correct import of newly transcribed precursor proteins in mitochondria [11,12].

Increased expression of *TOMM34* has been detected in various cancers including oral squamous cell [13] and hepatocellular carcinoma [14], ovarian [15], colorectal [16,17], bladder [18], and gastric cancer [19]. Such profound association with oncogenesis prompted to design a cancer vaccine that contains TOMM34-derived peptides for the treatment of colorectal cancer [20,21]. Moreover, *TOMM34* has been linked to neurodegenerative diseases [22,23] and immune response [24].

Altogether, these facts indicate that *TOMM34* is still under-characterized despite being associated with various human diseases. This can be achieved using multiple omics techniques which can reveal the most information regarding potential functions of genes and proteins [25]. Here we used CRISPR-Cas9 to generate *TOMM34*^-/-^ HepG2 cell line and comparatively profiled transcriptome, proteome, and metabolome for both wild type (WT) and knockout (KO) cells. An earlier study demonstrated that TOMM34^-/-^ mice grow normally, indicating that *TOMM34* is a nonessential gene [26]. Recently, it was shown that *TOMM34* knockdown in cell line models of oral squamous cell carcinoma leads to impaired growth and migration of cancer cells, as well as damage to mitochondria and increase in intracellular reactive oxygen species [27].

To our knowledge, the present study is the first attempt to investigate the functional capacity of *TOMM34* using a multi-omics strategy coupled with network-based system biology approach for in-depth analysis.

## 2. Materials and Methods

### 2.1. Cells Preparation

The HepG2 cell line was obtained from Sigma-Aldrich (Merck KGaA) and cultured in DMEM/F12 with an additional 10% fetal bovine serum and 100 units/mL penicillin/streptomycin (Gibco, Amarillo, TX, USA) in a humidified CO_2_-incubator under conditions 5% CO_2_, 37 °C. The medium was replaced every three days.

### 2.2. CRISPR-CAS9

At the first stage, we analyzed the target gene exons formed after splicing using Ensembl database (hg38 v103). The search for optimal protospacers was carried out using the E-CRISPR (http://www.e-crisp.org/E-CRISP/, accessed on 7 October 2021) and CRISPR-ERA (http://crispr-era.stanford.edu/, accessed on 7 October 2021) resources. The sequences were selected in the first exon to ensure the most efficient knockout. The target sequences were as follows 5′-GCCGTAGAGCGCGGAGGCCT, 5′-GCCGAGGCCTCCGCGCTCTA, 5′-GGAAGGTGTGCCGCACCACA, and 5′-CAGCCGTAGAAGGCATCAACAGG. The selected sequences of sgRNA spacers were synthesized as complementary DNA oligonucleotides for the subsequent production of duplexes. The resulting duplexes were ligated into the pX458 vector pre-cleaved with the BbsI enzyme (Addgene: #48138, Watertown, MA, USA), containing the Cas9 nuclease gene and guide RNA sequence (without a protospacer) under the control of the cytomegalovirus (CMV) and U6 promoters, respectively, for in vivo expression, as well as marker genes encoding GF) and ampicillin resistance.

At the second stage, plasmid DNA was transformed into *E. coli* XL1-Blue. The selection was carried out on the medium with ampicillin. The choice of colonies with an insert was performed using PCR with primers U6F and reverse primers specific to concrete spacer sequences. The correctness of cloning was confirmed by sequencing the plasmids with the U6F primer.

Next, HepG2 cells were transfected with the obtained plasmids using Genjector-U (Molecta, Russia). The cell culture was sorted to single cells in a 96-well plate on a cell sorter, selecting GFP-positive 2 days after transfection. After 3–4 weeks of incubation, clones were selected, seeded into the wells of a 6-well plate, and cultured. Gene knockout was confirmed by mass spectrometry.

### 2.3. Western Blotting

Cells were cultured under standard conditions and harvested by trypsinization upon reaching confluence of 70%. Cell pellets were rinsed twice with DPBS solution and lysed in 30 µL of RIPA buffer (150 mM NaCl, 1% Triton X-100, 0.5% sodium deoxycholate, 0.1% SDS, 50 mM Tris, and pH 8.0). Then, 10 µL of 4x Laemmli sample buffer containing DTT (60 mg/mL) was added to reach the final concentration of 1x. The samples were incubated at 95 °C for 15 min and stored at −80 °C until analysis.

The proteins were separated in 14% SDS-PAGE utilizing 50 µg of total protein per well and transferred onto nitrocellulose membrane (Bio-rad, Hercules, CA, USA). The membranes were incubated with EveryBlot blocking buffer (Bio-rad, Hercules, CA, USA) for 5 min and stained with primary and HRP-conjugated secondary antibodies for 1 h under rotation. Anti-TOMM34 (Santa-Cruz, #sc-101284) antibody was used for TOMM34 detection and anti-alpha-tubulin antibody (Abcam, ab7291) was used as loading control. ECL Blotting Substrate (Bio-rad, Hercules, CA, USA) was used to. develop the chemiluminescent reaction. The images were obtained with DNR LumiBis Gel Imaging System 3.2. and GelCapture software.

### 2.4. Transcriptome Profiling

The library preparation and transcriptome profiling were performed according to the protocol described earlier [28]. Briefly, total RNA was isolated from HepG2 cells with a RNeasy Mini Kit (Qiagen, Hilden, Germany) and extracted with a Dynabeads™ mRNA Purification Kit (Thermo Fisher Scientific, Waltham, MA, USA). Transcriptome was profiled by Illumina NovaSeq 6000 with a read length of 100 bp in three replicates for each sample.

Raw sequencing data were uploaded to the NCBI SRA (https://www.ncbi.nlm.nih.gov, accessed on 21 September 2021). The accession number is PRJNA765908 for both WT and KO cells: Datasets WT1, WT2, WT3, KO1, KO2, and KO3 can individually be downloaded via accession numbers SRR16071314, SRR16071313, SRR16071312, SRR22543760, SRR22543761, and SRR22543762, respectively.

### 2.5. Proteome Profiling

We processed 1 mln cells, diluted in 150 μL of 50 mM HEPES buffer. After three freeze-thaw cycles and centrifugation (4 °C, 15,000 rpm, 15 min), 135 μL of supernatant was collected and treated with 15 μL of the cocktail comprised from 1 mg of RapiGest™ SF Surfactant, 60 μL 0.5 M CAA, 20 μL 0.5 M tris(2-carboxyethyl)phosphine hydrochloride (TCEP), buffered by 20 μL 1 M HEPES. The reaction lasted for 30 min at 70 °C with vigorous shaking at 1500 rpm in darkness.

Enzymatic digestion was performed with trypsin, added to reduced and alkylated proteins to achieve the estimated ratio to the substrate as 1:40 (*w*/*w*). The trypsinolysis was incubated for 4 h at 37 °C. The digestion was quenched by trifluoroacetic acid (to pH < 3 finally), incubated for 30 min at 37 °C, and centrifuged at 13,000 g for 10 min. The supernatant (200 uL) with peptides was collected and transferred into the EvoTip to proceed HPLC-MS/MS analysis.

EvoSep One chromatography system connected to Orbitrap Exactive (Thermo Fisher Scientific, Waltham, MA, USA) mass spectrometer was used to separate and identify the peptides. Peptide separation was carried out on an EvoSep Endurance EV1106 column using 40 min chromatographic method.

Mass spectra were recorded in the positive ion mode. Data were obtained using the Orbitrap Exactive analyzer with a resolution of 70,000 (at m/z 400) for MS and 15,000 (m/z 400) for MS/MS scans. Higher energy collisional dissociation (HCD) was used to fragment the peptides. The signal threshold was established at 17,500 for an isolation window of 1 m/z. The first mass of HCD spectra was set to 100 m/z. The collision energy was set to 35%. Fragmented predecessors were dynamically excluded from targeting for 10 s. In addition, singly charged ions and ions with no defined charge conditions were excluded from triggering MS/MS scans. Three HPLC-MS/MS repetitions were accomplished for each sample.

Raw proteome data were uploaded to the Mendeley Data (DOI: 10.17632/yrmd8ygncn.1, https://data.mendeley.com/, accessed on 6 December 2022).

### 2.6. Metabolome Profiling

The protocol by O. Fiehn [29] was used for sample preparation. Briefly, intracellular metabolites were extracted with freeze-thawing cycles. At the next stage, the HepG2 low molecular weight fraction was sequentially extracted with a mixture of isopropanol, acetonitrile, and water (3:3:2, *v*/*v*/*v*), followed by a mix of acetonitrile and water (1:1, *v*/*v*). The supernatant was evaporated using SpeedVac vaporizer (Eppendorf, Hamburg, Germany), and oxidized with 10 μL of methoxyamine hydrochloride (20 mg/mL in pyridine) at 30 °C for 90 min on a thermoshaker (Eppendorf, Germany) at 1300 rpm. Next, the samples were derivatized by 91 μL of MSTFA with a mixture of fatty acid methyl esters at 37 °C for 30 min in thermoshaker at 1300 rpm. Samples were sent for GC×GC-MS acquisition after derivatization.

All the GC×GC-MS applications were performed using a 7890B chromatography system (Agilent Technologies, Santa Clara, CA, USA) and a time-of-flight mass spectrometer Pegasus BT 4D (LECO, Benton Harbor, MI, USA) equipped with an L-PAL3 autosampler (CTC Analytics AG, Zwingen, Switzerland).

All samples (1 μL) were injected through the glass liner (Restek, Bellefonte, PA, USA) in a split mode (50:1). Constant flow of 1 mL/min of helium (99.9999%) was maintained throughout the run. Then, the oven was heated to 60 °C, the equilibration time was 1 min, and the temperature ramped at the pace of 10 °C/minute to 280 °C with a hold time of 12 min. The 30 m long Restek Rxi-5Sil MS and 2 m long Restek Rxi-17Sil MS (Restek, Bellefonte, PA, USA) were used as the first- and second-dimension columns coordinately. The transfer line of the MS time was set at 280 °C, with a solvent delay of 350 s, while the ion source temperature was set to 250 °C. Spectra were assembled from 35–700 m/z at 70 eV electron ionization energy. The scan rate was set to two hundred spectra per second.

Raw metabolome data were uploaded to the Mendeley Data (DOI: 10.17632/yrmd8ygncn.1, https://data.mendeley.com, accessed on 6 December 2022).

### 2.7. Bioinformatics and Statistical Data Analysis

The quality of fastq files was checked using the FastQC [30]. Data were aligned to the reference transcriptome Ensembl cDNA v.103 using Hisat2 [31] for protein-coding transcripts from canonical chromosomes. Expression quantification in transcript per million units (TPM) was performed using Salmon [32]. We calculated gene expression by summing all TPMs for the respective transcripts.

Metabolome data were analyzed according to the protocol by Kiseleva et al. [33]. Summarily, the spectrum files were analyzed by ChromaTOF Tile (v. 5.51, LECO, Benton Harbor, MI, USA). We used the Fisher-ratio-based mode (F-ratio > 5), metabolite IDs were designated using The Human Metabolome Database (https://hmdb.ca/, accessed on 5 September 2022).

Raw proteome data were transformed into mgf files by MSConvert (v. 3) [34]. To process proteome data we used SearchGUI software [35] (v. 4.1.24, search engines were X!Tandem, MS-GF+, OMMSA) against SwissProt library of human canonical and alternatively spliced protein sequences in automatic mode [36]. The false discovery rate (FDR) cut-off for peptide-spectra matches, peptides, and proteins was ≤1%. We used the normalized spectral abundance factor (NSAF) for label-free quantification proteomics [37].

The R software environment was used for computations and visualization (ver. 4.1) [38]. We used ideal package [39] for differential expression analysis, EnhancedVolcano package for volcano plot (https://github.com/kevinblighe/EnhancedVolcano, accessed on 5 December 2022), ClusterProfiler [40] for geneset enrichment analysis and PCSF package [41] for de novo network enrichment. As a source of biological knowledge, we utilized community-curated Wikipathways database [42]. Networks were visualized with Cytoscape 3.9 [43].

## 3. Results

**Figure 1:**
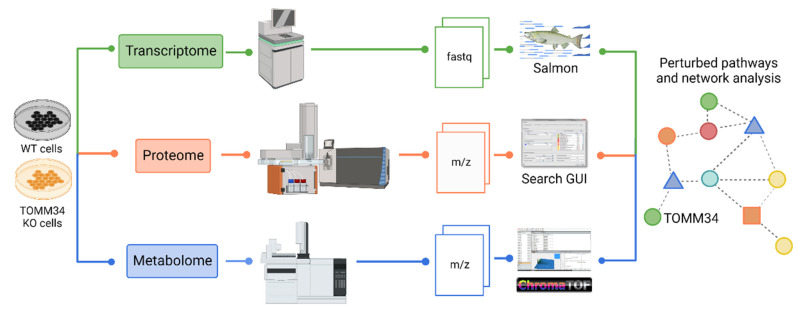
The overall workflow of the experiment to investigate the functional capacity of *TOMM34*.

The experiment setup is illustrated in Figure 1. To characterize *TOMM34* functions, we cultured both normal wild-type (WT) HepG2 cells and *TOMM34*^-/-^ HepG2 cells obtained using CRISPR-Cas9 technology. Knockout efficiency was verified by Western blotting (Supplementary Figure S1). Then, we performed comparative profiling of transcriptome, proteome, and metabolome (Supplementary Tables S1–S3) followed by systems biology analysis to identify significantly perturbed pathways and de novo subnetworks.

### 3.1. Transcriptomic Differences

**Figure 2:**
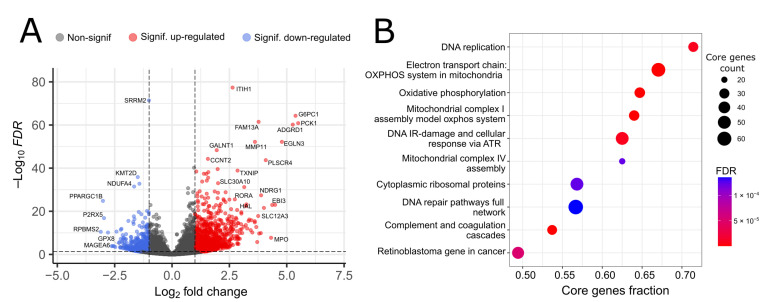
Transcriptomic profiling of HepG2 WT and *TOMM34* KO cells. (**A**) Volcano plot showing differentially expressed genes between WT and KO cells. FDR—false discovery rate. (**B**) Gene Set Enrichment Analysis of differentially expressed genes between WT and KO cells. Core genes is the subset of genes in the geneset contributing to the enrichment signal.

We used transcriptome profiling to quantify the expression of 16,541 genes in total between WT and KO cells. Differential expression analysis revealed 1639 genes with significantly altered expression, among which 481 genes were downregulated and 1158 genes were upregulated in KO cells (Figure 2A). In addition, we performed Gene Set Enriched Analysis with the resulting list of differentially expressed genes to reveal pathways most affected by *TOMM34* knockout (Figure 2B, Supplementary Table S4).

In conformity with the role of *TOMM34* in mitochondrial functioning, several associated pathways, such as “Electron transport chain, OXPHOS system in mitochondria” (FDR = 2.79 × 10^−8^) or “Mitochondrial complex IV assembly” (FDR = 2.83 × 10^−4^) were significantly downregulated upon *TOMM34* knockout. Recently, it was discovered that *TOMM34* stimulates mitochondrial OXPHOS and ATP production in metformin-treated liver cancer cells through interaction with ATP5B, a key subunit of mitochondrial ATP synthase, whose activity is critical for the functioning of the mitochondrial electron transport chain and connected processes [44]. Our data support these findings since we also observe decreased mitochondrial functioning after the knockout of *TOMM34*. We also observed downregulation of several DNA-related pathways, such as “DNA replication” (FDR = 2.55 × 10^−5^) and “DNA IR damage and cellular response via ATR” (FDR = 2.55 × 10^−5^), which cannot be comprehensively explained by the chaperone function of *TOMM34* only. Top upregulated pathways included several immunity-related gene sets, for instance, “Complement and coagulation cascades” (FDR = 4.37 × 10^−7^) and “Complement activation” (FDR = 6.5 × 10^−4^). Although *TOMM34* was recently found to be upregulated in activated CD4+ cells [24], its possible roles in immunity remain largely unexplored.

Differential gene expression results indicated alterations in several genes such as NDUFA4 (log_2_FC = −1.65). That gene encodes protein that participates in the mitochondrial complex IV assembly. The GSEA results showed that the oxidative phosphorylation (OXPHOS) system of the electron transport chain in mitochondria has significantly different genes. It also includes OXPHOS complexes disrupted in KO cells, such as the OXPHOS assembly system model of mitochondrial complex I (CI) and mitochondrial complex IV assembly (CIV). For example, differential gene expression results indicated alterations in NDUFA4 which is included in CIV. Suppression of this gene results in decreased CIV activity, which inhibits proliferation compared to non-knockout counterparts [45]. It is known that adenosine diphosphate (ADP) and adenosine triphosphate (ATP) are the main components of OXPHOS complexes. *TOMM34* plays an essential role in translocating nuclear-encoded mitochondrial proteins, including ADP/ATP carriers [46]. Thus, the downregulation of the gene may impact energy production and suppress the OXPHOS complexes [47]. In our analysis, we observed the suppression of *TOMM34* and, consequently, the downregulation of genes for the CI and CIV complexes. It was shown that intracellular ROS levels significantly increased after *TOMM34* knockdown, while reactive oxygen species (ROS) levels declined after *TOMM34* overexpression [27].

At the level of individual genes, the most significant decrease in expression was observed for RBPMS2 (log_2_FC = −3.11), which normally participates in the development and dedifferentiation of digestive smooth muscle cells [48], but has also been affiliated with the progression of several types of cancer [49]. Another significantly downregulated gene, PPARGC1B (log_2_FC = −3.01), encodes a protein PGC-1β, which is known as a master regulator of mitochondrial biogenesis [50]. A link exists between *TOMM34* and PGC-1β through transcriptional factor NRF1, which interacts with PGC-1β [51] and upregulates *TOMM34* [52]. Notably, for another downregulated gene, MAGEA6 (log_2_FC = −2.58), a role in tumor transformation and tumor progression was recently demonstrated [53]. This, together with the observed downregulation of GPX8 gene (log_2_FC = −2.27), which functions to combat oxidative stress [54], might explain the results from the previous study, where decreased proliferation and increased oxidative stress were observed for cancer cells after *TOMM34* knockdown [27].

Almost thrice more genes were upregulated, rather than downregulated, after *TOMM34* knockout. Of these, the biggest change in expression was observed for genes PCK1 (log_2_FC = 5.50) and G6PC1 (log_2_FC = 5.38). Notably, both of these genes are positively regulated by a transcriptional factor encoded by RORA gene [55,56], which is also upregulated upon *TOMM34* knockout (log_2_FC = 2.74). Concerning the association of *TOMM34* to cancer, another significantly upregulated gene, TXNIP (log_2_FC = 2.86), has been shown to perform tumor suppressor function in several cancer cell types [57].

### 3.2. Proteomic Differences

We then turned to comparative proteome profiling of HepG2 *TOMM34* KO and WT cells. A total of 1239 proteins were identified with 272 proteins identified only in HepG2 *TOMM34* KO cells and 444 proteins identified only in HepG2 *TOMM34* WT cells. We report that 523 proteins were identified in both cell types. Of note, *TOMM34* protein was detected in all WT replicates and was absent in all KO replicates, thus indicating the success of gene knockout.

**Figure 3:**
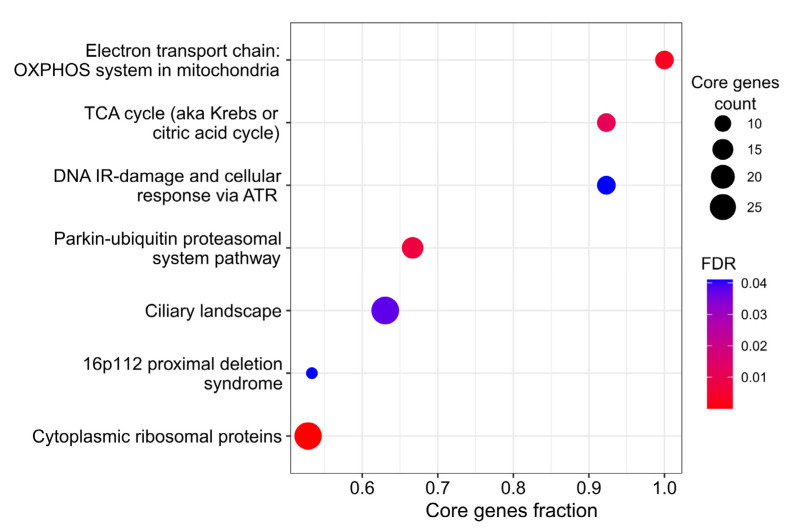
Proteomic profiling of HepG2 WT and *TOMM34* KO cells. Gene Set Enrichment Analysis: pathways perturbed in *TOMM34* KO cells compared to WT cells. Core genes is the subset of genes in the geneset contributing to the enrichment signal.

To detect cellular processes perturbed by *TOMM34* knockout, we run GSEA for protein abundance fold-changes. The results are presented in Figure 3 and Supplementary Table S5. Among the significantly deregulated pathways with a high proportion of proteins of interest, we found the electron transport chain (FDR = 0.002) and Krebs cycle (FDR = 0.02). Interestingly, the disbalance of the Krebs cycle appears in the proteomic data and not at the transcriptome level. Considering the role of *TOMM34* in mitochondrial protein import, it is possible that *TOMM34* knockout interferes with the abundance of several key proteins of the Krebs cycle, while mRNA levels of these proteins remain relatively stable. The activity of the Krebs cycle leads to the production of electron carriers such as NADH and FADH2, which are necessary to support the electron transport chain activity [58]. Accordingly, the deregulation of the Krebs cycle may play a part in the observed downregulation of mitochondrial respiration. This conforms with the aforementioned transcriptomic results and suggests that the most affected organelle is mitochondria, which is in agreement with current knowledge about *TOMM34* cellular role.

The deregulation of the ionizing radiation-induced damage to DNA was also previously observed in transcriptomics data. This suggests that *TOMM34* may be indirectly associated with ATR, a PI3K-like protein kinase that plays a key role in regulating DNA damage responses. It was shown that ATR has an antiapoptotic activity at mitochondria in response to UV damage [59].

The parkin–ubiquitin proteasome system is another pathway that is affected more at the protein level rather than at the transcript level. This pathway represents one of the major protein degradation pathways, in which parkin ubiquitinates a wide variety of cytosolic and outer mitochondrial membrane proteins [60]. Remarkably, parkin activity is directly connected to the mitochondrial state through PTEN-induced putative kinase protein 1 (PINK1) [61]. This protein kinase is normally transported to mitochondria via TOM complex and subsequently degraded [62]. However, when mitochondrial functioning is disrupted, PINK1 accumulates on mitochondrial outer membrane and recruits parkin, which activity can lead to the selective elimination of the mitochondrion (mitophagy) [63]. Perturbation of the parkin–ubuquitin proteosomal pathway might provide clues to the association of *TOMM34* with neurodegenerative diseases [22,23].

Overall, the results of proteomic profiling support and extend the list of pathways associated with *TOMM34* functioning obtained from the transcriptome analysis.

### 3.3. Metabolic Differences

We measured metabolomic differences between KO and WT *TOMM34* HepG2 cells using GC×GC-MS acquisition. A total of 73 metabolites were identified with 54 being significantly different between the two conditions (46 upregulated and 8 downregulated by *TOMM34* knockout). The most significantly upregulated metabolites included inosine (log_2_FC = 4.2), glycerol and guanosine (both log_2_FC = 3.7), xanthine (log_2_FC = 3.2), L-homoserine and ornithine (both log_2_FC = 3.1). The most significantly downregulated metabolites included citric acid (log_2_FC = −2.6) and fructose 6-phosphate (log_2_FC = −1.0). The full table of identified metabolites is given in Supplementary Table S3.

From single-metabolite analysis, we then turned to the identification of pathways perturbed by *TOMM34* knockout using the metabolomics data. We utilized RaMP-DB [64] to perform pathway enrichment analysis with the list of differential metabolites serving as an input. An attractive feature of RaMP-DB is its ability to improve the interpretability of pathway analysis results by grouping enriched pathways into clusters.

**Figure 4:**
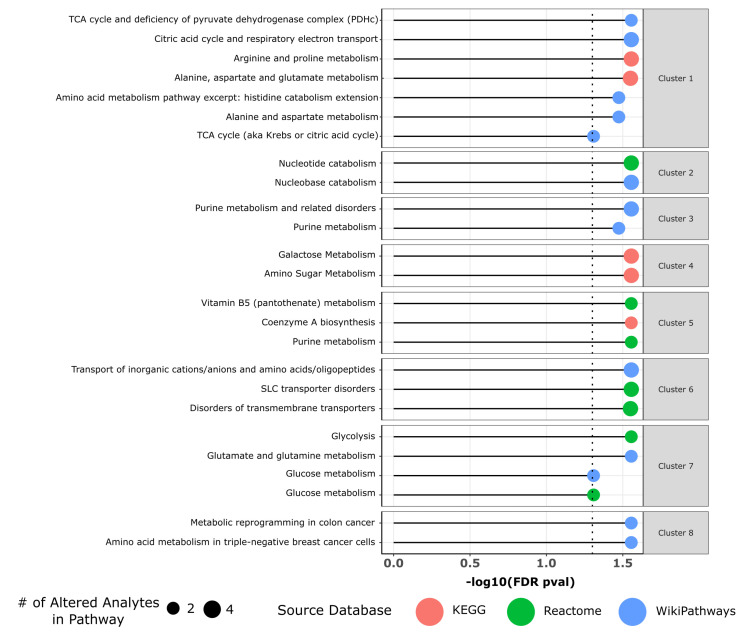
Results of the metabolic pathway enrichment analysis by RaMP-DB [64]. Significantly enriched metabolic pathways from KEGG, Reactome, and WikiPathways are organized into groups.

A total of 40 pathways with FDR < 0.05 were identified with 25 pathways grouped into 8 clusters and 15 pathways remaining unassigned to any cluster (see Supplementary Table S6 for the full list). The results are presented in Figure 4.

The first pathway cluster consisted of cellular processes such as metabolism of various amino acids, citric acid cycle, and respiratory electron transport. This finding may indicate that deregulated mitochondrial transport due to *TOMM34* knockout results in altered amino acid metabolism, since mitochondria are known to play this important biosynthetic role in addition to the canonical ATP generation [65].

Pathway cluster #3 and cluster #5 corresponded to the altered purine metabolism. The observed changes in purine metabolism are also connected with another affected process, nucleotide catabolism (cluster #2). High cellular purine demands in extensively proliferating cells lead to the upregulation of the de novo biosynthetic pathway, which requires metabolic precursors including such amino acids as glutamine, glycine, and aspartic acid [66]. The metabolism of these amino acids is also affected by *TOMM34* knockout, as we reported previously. Detected alterations in purine metabolism may serve as a mechanical explanation of the previous finding of deregulated DNA replication from analysis of transcriptomics data.

Pathway cluster #4 included galactose and amino sugar metabolism, while cluster #6 was associated with altered transmembrane transport, likely the source of observed changes in metabolism. Cluster #7 included altered glucose metabolism, as a primary carbon source for biosynthesis. Finally, cluster #8 indicated the role of metabolic alterations in colon and breast cancer, thus providing links to the observed upregulation of *TOMM34* in various tumors.

Analysis of metabolomics data revealed a lot of cellular processes which were affected by the *TOMM34* knockout, which is not surprising given the vital role of mitochondria in maintaining cellular metabolism.

### 3.4. De Novo Network Enrichment

Although geneset enrichment analysis is the common way to identify deregulated molecular mechanisms, it is limited to already known signaling or metabolic pathways. The alternative approach is to use the so-called de novo network enrichment methods which map the available data to the global network of gene, protein, and metabolite interactions and extract dense subnetworks enriched for entities that are implicated in the phenotype of interest.

We tried to take advantage of both approaches by combing reliable high-throughput protein–protein interactions with established biological knowledge. We merged HumanNet-FN v3 [67], a functional gene network encompassing associations between human genes derived via computational and experimental approaches, with the network of human metabolism [68], which includes all molecular interactions reported in the KEGG database [69]. The final combined network consisted of 22,396 genes and metabolites and 1,026,783 interactions between them.

We used this global network and the multi-omics data from WT and KO cells as input to the prize-collecting Steiner forest (PCSF) algorithm [41] to identify subnetworks enriched with perturbed genes, proteins and metabolites. Biological interpretation of these subnetworks may give clues on the functional role of *TOMM34* gene. This PCSF algorithm aims to find a forest from the global graph with a maximum sum of so-called prizes (absolute value of gene or metabolite log_2_ fold changes) while penalizing for the inclusion of hubs. It is important that the PCSF method is not required to consider all the obtained omics data but is able to include non-quantified nodes from the global network to establish connections between the nodes with high prizes. Thus, the extracted subnetwork has more chances to represent the actual molecular mechanisms perturbed by the *TOMM34* knockout.

The full solution obtained by the PCSF algorithm consisted of 1262 nodes (1215 genes and 47 metabolites) connected by 7237 relations in the global network. Similar to the approach described by Soltis and coauthors [70], to simplify the interpretation of the resulting network, we utilized the community clustering algorithm [71] to break the full network into 67 smaller subnetworks. For each subnetwork, we performed pathway enrichment analysis and calculated prize density as the sum of prizes divided by a fraction of the subnetwork size relative to the global network.

**Figure 5:**
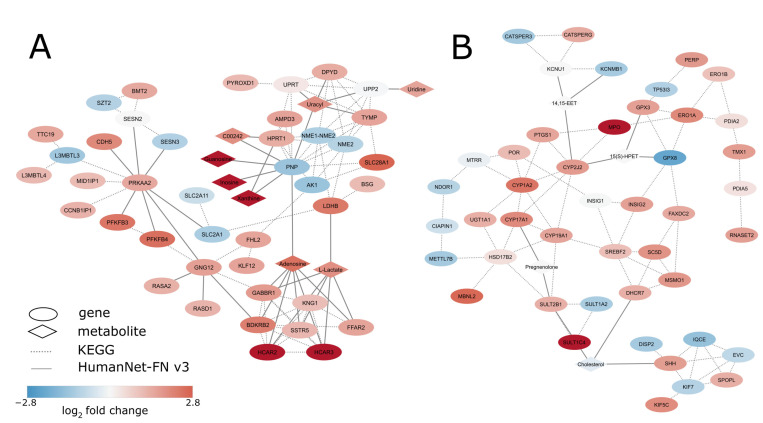
Results of de novo network enrichment analysis: Subnetworks enriched with genes, proteins, and metabolites perturbed in TOMM34 KO cells compared to WT cells. (**A**) Subnetwork #18, related to purine and pyrimidine metabolism. (**B**) Subnetwork #41, related to biotransformation, including oxidation by cytochrome P450.

The obtained list of all subnetworks can be found in Supplementary Table S7. A total of 46 subnetworks with enrichment FDR < 0.05 were ranked according to prize density. The composition of the first two subnetworks with the highest prize density, #18 (Pyrimidine metabolism, fluoropyrimidine activity, purine metabolism) and subnetwork #41 (Biotransformation phase I and II, oxidation by cytochrome P450, classical pathway of steroidogenesis with glucocorticoid and mineralocorticoid metabolism) (Figure 5A,B).

The subnetwork with the highest prize density, #18, was related to purine and pyrimidine metabolism, and most nodes in the network were upregulated (Figure 5A). This is interesting considering previously detected downregulation of DNA replication and DNA repair pathways. A possible explanation involves PNP gene, which encodes an enzyme central to the purine salvage pathway and which is downregulated in KO cells (log_2_FC = −1.42). Downregulation of the PNP gene leads to the accumulation of its substrates such as guanosine and inosine [72], which is consistent with our data (Figure 5A). Accumulation of PNP substrates, most notably deoxyguanosine, can lead to severe inhibition of DNA replication and repair processes [73]. Furthermore, increased expression and activity of PNP is commonly observed in cancer [74]. This is not surprising considering elevated levels of nucleotide metabolism are necessary for cancer cells [75].

Other primarily metabolism-related subnetworks, for instance, aforementioned subnetwork #41 (Oxidation by cytochrome P450, Steroidogenesis with glucocorticoid and mineralocorticoid metabolism, Figure 5B), subnetwork #9 (Transsulfuration and one-carbon metabolism, Drug induction of bile acid pathway, Amino acid metabolism), subnetwork #4 (Glycolysis and gluconeogenesis, Clear cell renal cell carcinoma, Glycolysis in senescence), and subnetwork #25 (Urea cycle and metabolism of amino groups, Amino acid metabolism, Methionine de novo and salvage pathway) were also frequently perturbed upon *TOMM34* knockout. Deregulation of these pathways can be explained by the central role of mitochondria in metabolism, particularly in nucleotide and amino acid metabolism [76]. As reported previously, *TOMM34* knockout leads to the impaired import of some of the mitochondrial proteins, some of which may play primary or auxiliary function in supporting metabolic processes in mitochondria [10].

Furthermore, we observe the deregulation of multiple major cellular signaling pathways such as subnetwork #15 (Apoptosis, Apoptosis modulation and signaling, Hippo-Merlin signaling dysregulation), subnetwork #14 (PI3K/Akt signaling pathway, Therapy-induced HIF1 survival signaling, Mesothelioma), and subnetwork #11 (Notch signaling, MAPK signaling pathway, Canonical and noncanonical Notch signaling). Previously, it was shown that ERK/MAPK signaling is inhibited in osteosarcoma cells after *TOMM34* knockdown [27]. It is well known that signaling pathways in cells are significantly interconnected [77], so the observed effect might be related to the initial perturbation of ERK/MAPK signaling.

Several subnetworks include pathways related to cancer, such as already mentioned subnetwork #4 and subnetwork #14, as well subnetwork as #13 (STAT3 signaling and glioblastoma signaling). This is rather expected since previously discussed global cellular signaling pathways frequently crosstalk with signaling pathways driving oncotransformation [78].

## 4. Discussion

Mitochondria are vital organelles with a key role in cell energy production and metabolism. As a part of TOM complex, TOMM34 is thought to play an important role in maintaining mitochondria function. Although *TOMM34* functions are not yet fully clarified, it is likely that they will manifest on all layers of molecular organization, including transcriptome, proteome, and metabolome.

Therefore, a comprehensive functional characterization of *TOMM34* requires multi-omics profiling. To our knowledge, this is the first attempt to study the functional capacity of *TOMM34* using a multi-omics strategy coupled with a network-based system biology approach for an in-depth analysis.

Our study revealed that *TOMM34* knockout perturbed various cellular processes. Some of these processes are clearly related to the mitochondria functioning including oxidative phosphorylation, citric acid cycle, and assembly of mitochondrial complexes I and IV. This is in line with recent findings by Huang and coauthors, who reported that *TOMM34* participates in maintaining the mitochondrial shape and regulates the intracellular reactive oxygen species level [27].

While some pathways were found to be deregulated by analysis of a single omics layer, several cellular processes were independently confirmed by multiple types of omics data. For example, the electron transport chain and citric acid cycle were detected as significantly enriched by all three omics layers, thus increasing the reliability of the analysis. We found UV-induced DNA damage to be supported by both transcriptomics and proteomics profiling. There were also cases when the pathway detected via analysis of a single omics layer was indirectly confirmed by another omics. According to the metabolome analysis, *TOMM34* knockout resulted in altered purine metabolism, while transcriptomics data showed significant enrichment of DNA replication pathway. Thus, we speculate that since *TOMM34* is upregulated in tumor cells with increased demand in nucleotide synthesis due to accelerated proliferation, the observed perturbation of DNA replication may be related to alterations in purine metabolism due to *TOMM34* knockout.

The usage of de novo network enrichment is the distinctive feature of the conducted system biology analysis, preventing the potential bias towards already known pathways. Extraction of previously uncharacterized perturbed subnetworks allowed us to detect other cellular processes implicitly associated with *TOMM34* including NOTCH-, MAPK-, and STAT3 signaling, frequently deregulated in cancers. This may give clues to *TOMM34* complex and possibly contextual effects on tumorigenesis, which is confirmed by the experimental fact that *TOMM34* increases the activity of ERK1/2 and MEK1/2 in HPV-positive oral squamous cell carcinoma cells but not in HPV-negative [27]. Moreover, this type of analysis enables us to perform true multi-omic integration via projecting available data onto a global network connecting genes, proteins, and metabolites. Since there are many crosstalks between various mitochondria-associated processes affected by *TOMM34* (glycolysis, amino acid metabolism, purine metabolism, etc), the aforementioned network-based approach is capable of taking full advantage of the availability of multi-omics data.

While the experimental part was performed using HepG2 cells, this does not imply that the same results will be reproduced for other lines. Moreover, one can consider utilizing other system biology algorithms for de novo network enrichment, such as KeyPathwayMineR [79] or SmCCNet [80]. However, it was shown that GSEA can perform even better than complicated topology-based methods [81]. Another direction to extend our work is to perform the gain-of-function study by transcriptional activation of *TOMM34* expression followed by omics profiling. These issues likely will be in the focus of subsequent investigations.

## 5. Conclusions

The aim of our study was to characterize functional role of *TOMM34*. In brief, we found that *TOMM34* affects various processes including oxidative phosphorylation, citric acid cycle, and metabolism of purine and several amino acids.

## Data Availability

Raw transcriptome data files are publicly released on the NCBI SRA (https://www.ncbi.nlm.nih.gov, accessed on 6 December 2021). The accession number is PRJNA765908 for both WT and KO cells. Raw proteome and metabolome data are publicly released on the Mendeley Data (https://data.mendeley.com/, accessed on 6 December 2022). DOI: 10.17632/yrmd8ygncn.1.

## Supplementary Materials

The following supporting information can be downloaded at: https://www.mdpi.com/article/10.3390/biology12020198/s1, Table S1: transcriptomics profiling, Table S2: proteomics profiling, Table S3: metabolomics profiling, Table S4: transcriptomics enrichment, Table S5: proteomics enrichment, Table S6: metabolomics enrichment, Table S7: de novo network enrichment; Figure S1: Western blot result: TOMM34 protein level in WT and KO HepG2 cells.
