# Supplementary figures and images for: Exploiting Multi-Omics Profiling and Systems Biology to Investigate Functions of TOMM34
Source: Biology (Basel). 2023 Jan 28;12(2):198. doi: 10.3390/biology12020198 (PMC9952762; doi:10.3390/biology12020198)

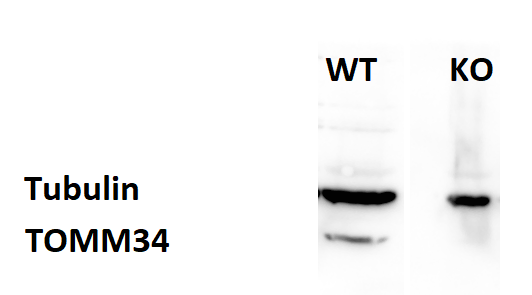

Supplement: Supplementary file 1 [file biology-12-00198-s001.zip › Figure S1.png]
